# Microsatellite characterization of *Plasmodium vivax* in pregnant women on the Thai–Myanmar border

**DOI:** 10.1186/1475-2875-11-S1-P137

**Published:** 2012-11-09

**Authors:** Supinya Thanapongpichat, Mallika Imwong, Rose McGready, Nicholas PJ Day, Francois Nosten, Georges Snounou, Nicholas J White

**Affiliations:** 1 Department of Clinical Tropical Medicine, Faculty of Tropical Medicine, Mahidol University, Bangkok, Thailand; 2 Department of Molecular Tropical Medicine and Genetics, Faculty of Tropical Medicine, Mahidol University, Bangkok, Thailand; 3 Shoklo Malaria Research Unit, Mae Sot, Tak, Thailand; 4 Mahidol-Oxford Tropical Medicine Research Unit, Faculty of Tropical Medicine, Mahidol University, Bangkok, Thailand; 5 Centre for Clinical Vaccinology and Tropical Medicine, Churchill Hospital University of Oxford, Oxford, UK; 6 Institut National de la Santé et de la Recherche Médicale, Unité Mixte de Recherche S 945,Paris, France; 7 Université Pierre & Marie Curie, Faculté de Médecine Pitié-Salpêtrière, Paris, France

## Background

*Plasmodium vivax* infections in pregnant women are associated with low birth weight and anemia. Genotyping of *P.vivax* is useful to study *P. vivax* in pregnancy, though it is still remains challenging to distinguish between relapse from hypnozoite stage and recrudescence from blood stage following treatment. A genetic investigation of *P.vivax* in pregnancy on the Thai-Burmese border, comparing the genotype between at follow up with the day of admission for pregnant patients and non pregnant patients was undertaken in this study.

## Materials and methods

One hundred and sixteen blood samples infected with *P.vivax* were isolated from 18 pregnant women with ≥2 episodes obtained from whole blood (12 women had 2 reappearances, 4 and 2 women had 3 and 4 reappearances, respectively) and 18 non-pregnant women with 2 consecutive episodes collected on dried blood spot. All samples were genotyped with eight microsatellite markers. Analyses were performed for genetic diversity, multiplicity of infection (MOI), and comparison of genotypes for individual episodes detected in samples.

## Results

Eight microsatellite loci, 6 to 15 alleles were found at each locus. The mean number of alleles per locus was 1.40 and 1.17 (*p*<0.001) for pregnant and non-pregnant patients, respectively. The overall mean expected heterogygosity (*He*) was 0.845 in both groups. In pregnant patients, the multiplicity of infection (MOI) was 1.85 while it was 1.44 (*p*=0.028) in non-pregnant patients.

The greater number of minor alleles in pregnant patients may either the nature of the sample.

Combined genetic data from days of follow and day of admission showed that genotypes were different 57 % (25/44) of those pregnant patients and 58 % (21/36) of non-pregnant patients (*p*=0.891).

## Conclusions

This study confirmed that different *P.vivax* genotypes were found during follow up when compared to day of admission in both groups.

